# Progress Analysis of Personalized Antiplatelet Therapy in Patients with Coronary Heart Disease Undergoing Interventional Therapy

**DOI:** 10.31083/j.rcm2512462

**Published:** 2024-12-25

**Authors:** Ji-tong Yang, Qiu-juan Zhang, Hua Li, Ming-wei Liu

**Affiliations:** ^1^Department of Clinical Medicine, Kunming Medical University, 651106 Kunming, Yunnan, China; ^2^Department of Emergency, The First Affiliated Hospital of Kunming Medical University, 650032 Kunming, Yunnan, China; ^3^Department of Emergency, The Third People’s Hospital of Yunnan Province, 650011 Kunming, Yunnan, China; ^4^Department of Emergency, People’s Hospital of Dali Bai Autonomous Prefecture, 671000 Dali, Yunnan, China

**Keywords:** interventional treatment, coronary heart disease, personalized therapy, antiplatelet therapy

## Abstract

Coronary atherosclerosis (or coronary heart disease [CHD]) is a common cardiovascular disease that seriously damages human health. Percutaneous coronary stent implantation represents the primary treatment option for severe CHD in clinical practice; meanwhile, dual antiplatelet therapy (DAPT) is widely used to reduce the risk of postoperative thrombosis. Although the mechanisms of action of the two most commonly used antiplatelet drugs, aspirin and clopidogrel, remain unclear, clinical studies have shown that some patients are susceptible to stent thrombosis—antiplatelet resistance (high on-treatment platelet reactivity [HTPR])—despite using these drugs. Therefore, screening for HTPR and formulating personalized antiplatelet therapies is necessary. Ticagrelor, indobufen, and rivaroxaban are the most common and safe antiplatelet drugs used in clinical practice, with broad application prospects. This review summarizes the mechanisms of action of existing antiplatelet drugs, reasons for personalized treatment, screening of antiplatelet reactions, and development of novel antiplatelet drugs.

## 1. Introduction

At present, coronary heart disease (CHD) is the leading cause of mortality in industrialized societies [1]. The incidence of CHD is high among middle-aged and older individuals [2]. As the age of the population and the trend of unfavorable diets and lifestyles increases, the incidence of CHD is rising concurrently annually [3, 4]. Currently, treatment strategies for CHD primarily include drug therapy, interventional therapy, and bypass surgery, among which percutaneous coronary intervention (PCI) is particularly important owing to its advantages of high efficiency and safety [5].

**Fig. 1.**
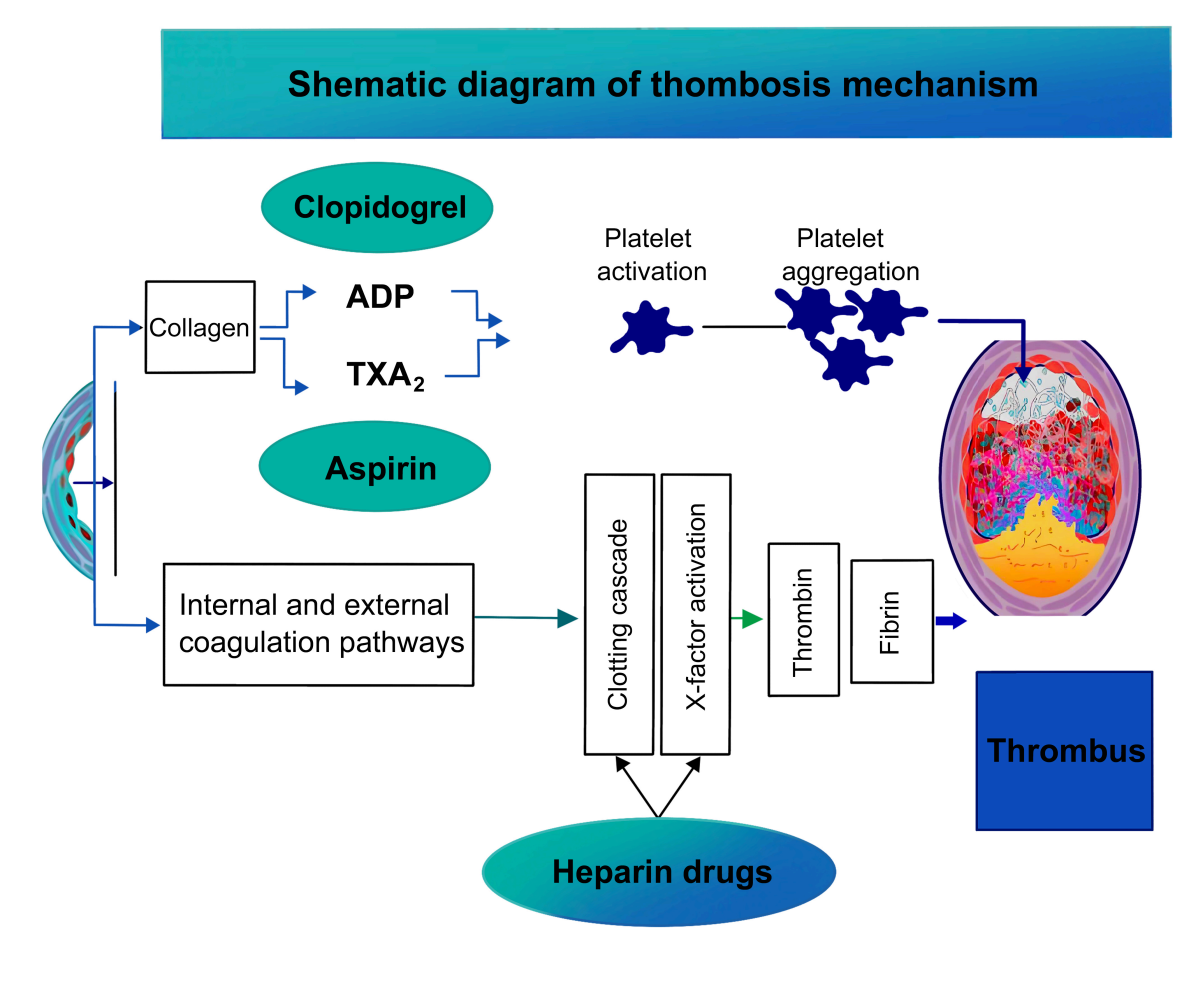
**Schematic diagram of the mechanism of thrombogenesis**. TXA_2_, thromboxane A2; ADP, adenosine diphosphate.

PCI opens narrowed or blocked blood vessels, effectively restoring the blood flow rate in the lumen, improving the blood supply to the myocardium, and alleviating myocardial damage [6]. However, stent implantation inevitably causes damage to the vascular wall, leading to abnormal inflammatory reactions, activation of platelets and coagulation components, and proliferation of vascular smooth muscle cells. These pathological processes eventually result in stent thrombosis (Fig. 1). Therefore, there are risks of coronary restenosis, no-reflow, and stent thrombosis after PCI, with varying degrees affecting the efficacy and long-term prognosis of PCI treatment. After administration, some anti-thrombotic drugs can be rapidly absorbed by the human body, inhibiting platelet agglutination and preventing thrombosis and restenosis. However, some patients may develop drug resistance owing to high on-treatment platelet reactivity (HTPR), which seriously affects therapeutic efficacy. Therefore, screening patients with HTPR [7, 8] is necessary to formulate personalized treatment strategies for preventing and treating thrombosis [9].

## 2. Mechanism of Antiplatelet Therapy

The narrowing and blockage of coronary arteries are direct causes of CHD, which restricts blood flow to the heart. As the viscosity of blood increases in narrow vessels, its flow becomes slower, resulting in the formation of blood clots on the inner walls of blood vessels [10, 11]. A disorder in the blood clotting mechanism facilitates the formation of blood clots and causes more damage to artery walls, whereas platelet aggregation exacerbates this process. Some studies have shown that vasodilators positively affect coronary artery stenosis [12, 13]. Platelet activation plays a key role in arterial thrombosis and atherosclerosis and contributes to the development of CHD [14, 15]. Cyclooxygenase inhibitors can be used to block platelet activation, thereby preventing thrombosis. Cyclooxygenase in platelets converts arachidonic acid to thromboxane and prostaglandins. Thromboxane promotes platelet coagulation to a certain extent, leading to thrombosis.

Platelets play a role in clearing cell debris and other substances in blood circulation. When stimulated, they rapidly gather and adhere to blood vessel walls, leading to a series of complex biochemical changes that eventually cause thrombosis [16]. Thrombin is a key factor that stimulates platelets to release soluble molecules and participates in platelet activation and aggregation. Adenosine diphosphate (ADP), a signaling molecule for intracellular energy transfer, has also been shown to stimulate platelet activation [17, 18]. Common antiplatelet drugs mainly include cyclooxygenase inhibitors, ADP receptor antagonists, and phosphodiesterase inhibitors, and these drugs act on these platelet-stimulating factors or their corresponding receptors to inhibit platelet activation and prevent platelet aggregation [19].

## 3. Reasons for Personalized Treatment

After PCI, patients have varying responses to antiplatelet therapy, a phenomenon called variability of platelet reactivity (VPR) [20]. For patients with high platelet reactivity in coronary heart disease, platelet activity may not be effectively inhibited after antiplatelet therapy with clopidogrel or aspirin, resulting in clopidogrel or aspirin resistance, leading to poor treatment efficacy and a higher risk of recurrent coronary artery thrombosis [21, 22]. Further, genetic, cellular, and other factors result in high platelet reactivity. Studies have shown that abnormal activation of the P2Y12/P2Y1 pathway, accelerated platelet renewal, and increased ADP exposure are important cellular mechanisms contributing to high platelet reactivity. In addition, conventional drug treatment can lead to drug resistance and high platelet reactivity, both of which are unfavorable for treatment [23, 24, 25, 26].

Aspirin inhibits the activity and metabolic pathways of platelets, thereby reducing thrombosis. Clopidogrel prevents platelet aggregation by blocking ADP receptors on the surface of platelets. However, some patients undergoing clopidogrel treatment may develop antiplatelet resistance and acute stent thrombosis, which delay recovery. The combination of ticagrelor and aspirin has gradually replaced emergency PCI for treating acute coronary syndrome [27].

## 4. Methods for Assessing Platelet Function

Platelet function can be assessed using several methods, such as light transmission aggregometry (LTA), platelet function analyser (PFA)100, vasodilator-stimulated phosphoprotein (VASP), VerifyNow P2Y12, multi-electrode array (MEA), and thromboelastography (TEG).

### 4.1 LTA

LTA is considered the gold-standard method for assessing platelet activity [28] and is based on the principle that platelet aggregation increases light transmission. When platelet-rich plasma is stimulated with specific inducers, platelets rapidly aggregate and form microclots. These platelets interact with other plasma components, resulting in a significant decrease in plasma turbidity and an increase in light transmission. Consequently, substances in the plasma can be easily visualized through fluorescence microscopy. Changes in light transmittance can be measured to determine platelet activity, providing important information for clinical diagnosis and treatment [29]. In a study, receiver operating characteristic (ROC) curve analysis showed that the maximum platelet agglutination rate induced by 5 µmol/L ADP was >46%, which validated HTPR. Additionally, stepwise regression analysis showed that high platelet counts were significantly associated with acute myocardial infarction [30].

### 4.2 PFA100

PFA100 is a novel *in vitro* system for detecting platelet dysfunction, which comprises a microcomputer-controlled device and a disposable test kit containing a bioactive membrane. The device aspirates blood from a sample reservoir under a constant-pressure vacuum and at a high shear rate through a capillary tube and the membrane. The membrane is coated with collagen, adrenaline, or ADP, which stimulates the platelets to adhere, activate, and aggregate to gradually form a stable plug, occluding the aperture and stopping blow flow—the time required to form the plug is recorded as the “closing time” [31, 32].

### 4.3 VASP

The VASP assay is used to examine the effects of the P2Y12 receptor by monitoring its activity in platelets. Activation of the P2Y12 receptor inhibits the activity of vitamin K antagonists. The VASP assay is widely used in clinical practice to evaluate the effects of drugs, such as clopidogrel, on platelets [33]. Quantitative analysis of phosphorylated VASP can more accurately indicate whether a patient is experiencing platelet inhibition or antiplatelet resistance.

According to the American College of Cardiology guidelines, patients with a probability of developing a major adverse cardiovascular event within 6 months of PCI >50% when using the area under the curve (AUC) method are considered to have HTPR. Treatment with antiplatelet drugs, such as clopidogrel, is continued if the negative predictive value is 100 [34].

### 4.4 VerifyNow P2Y12

The VerifyNow platelet function analyzer allows rapid assessment of the response to antiplatelet drugs (such as P2Y12 receptor inhibitors and aspirin). The VerifyNow aspirin system is a highly sensitive assay used to evaluate the inhibitory effects of aspirin on the arachidonic acid (AA) and thromboxane A2 (TXA_2_) pathways [35]. The system was designed to gain in-depth insights into the mechanisms underlying the antiplatelet activity after administering aspirin. In this assay, AA is used as a key platelet activator. If aspirin is effective, the cyclooxygenase-1 (COX-1) protein will be inhibited, preventing the conversion of AA to TXA2 and consequently blocking platelet activation. Therefore, the VerifyNow aspirin system can be used to assess platelet function and reactivity, such as platelet aggregation ability [36].

The system not only allows accurate assessment of the antiplatelet effects of aspirin but also helps to understand the complex pathways and mechanisms involved in platelet activation. Targeting these pathways or mechanisms may help understand the mechanism through which aspirin exerts its antiplatelet effects, thereby providing novel avenues for expanding the clinical application of this classic drug [37].

### 4.5 MEA

MEA examines platelet function in whole blood [38]. This method involves the placement of electrodes in a container of whole blood. These electrodes maintain blood flow and provide a platform for platelets to aggregate. Subsequently, certain inducers are added to the blood, which triggers the platelets to adhere to the electrodes and aggregate; platelet aggregation leads to changes in electrical impedance [39]. These changes can be recorded and analyzed to assess platelet activity and the extent of platelet aggregation accurately.

In particular, an increase in electrical impedance exceeding 48 µV/cm indicates HTPR, an important risk factor for death in many patients.

### 4.6 TEG

TEG was developed by Dr. Hellmut Hartert (University of Heidelberg, Germany) in 1948 and is widely used to measure platelet function based on coagulation and fibrinolysis in clinical settings [40]. This method adds a limonized or heparinized whole blood sample to an oscillating cup containing a detection pin in its center. After a coagulant is added, the amplitude of the oscillating movement is recorded using the pin connected to a torsion line. The main indicators are reaction time (R) and maximum amplitude (MA) of the curve. R reflects the blood coagulation rate, whereas MA demonstrates the extent of thrombosis. The higher the MA, the higher the risk of thrombosis.

Additionally, TEG assesses dynamic changes in blood clot formation, strength, and degradation, providing insights into coagulation and fibrinolysis. It also enables quantitative analysis of platelet function, which may help determine the precise role of platelets in clotting [41].

With advancements in medical research, TEG has become an important tool for monitoring patients’ coagulation during cardiac surgery or detecting bleeding/coagulation disorders in patients with various cardiovascular diseases [42].

However, all of the methods mentioned above possess certain advantages and disadvantages. Indeed, HTPR measured using any of these methods has a negative trend of high and a positive trend of low, which may be related to the role of various stimulating factors in the body. However, all these methods are performed *in vitro *using a single inducer. Meanwhile, other factors may influence the antiplatelet response in addition to platelet hyperreactivity. Details regarding the six methods mentioned above are provided in Table 1 (Ref. [43, 44, 45, 46, 47, 48]).

**Table 1.** **Characteristics of various methods used to assess antiplatelet resistance**.

Methods for assessing antiplatelet resistance	Characteristics	Specificity	Sensibility
LTA	Considered the “gold standard” treatment; however, it also possesses some limitations, such as tedious sample pretreatment, poor repeatability, requires a large sample volume, is time-consuming, and has a high technical difficulty [43].	Strong	Strong
VerifyNow P2Y12	A simple, rapid (requires only 5 minutes) method whose results are fairly consistent with those of LTA. It has a high predictive value for major cardiovascular events (MACEs), but clinical trials are expensive [44].	Strong	Strong
VASP	This method is convenient and rapid, but its agreement with LTA (r = 0.688) is low [45].	Strong	Strong
PFA100	A rapid and simple test that can be performed using whole blood at the bedside and only requires a small sample volume; however, only epinephrine and collagen can be used to coat the membrane, which limits the use of antiplatelet drug efficacy observations [46].		
MEA	This simple method requires whole blood, but the detection cost is high, and the sample cannot be used if the platelet content is low [47].	Strong	Strong
TEG	Offers a wide range of applications and has a high predictive value for MACEs [48].	Strong	Strong

LTA, light transmission aggregometry; VASP, vasodilator-stimulated phosphoprotein; MEA, multi-electrode array; TEG, thromboelastography; PFA100, platelet function analyser 100.

## 5. Research Progress of Novel Antiplatelet Drugs

### 5.1 Ticagrelor

ADP is an important metabolite in the human body. It functions by recognizing P2Y receptors on the surface of platelets. These receptors are involved in platelet activation, which allows platelets to aggregate and form blood clots. Under physiological conditions, the surface of platelets contains only a small amount of these receptors. However, when platelets are stimulated under pathological conditions, they release a large amount of ADP that binds to P2Y receptors, eventually inducing platelet activation and aggregation [49, 50].

P2Y receptors include P2Y1 and P2Y12. Studies have shown [51, 52] that P2Y12 expression is significantly higher than P2Y1 expression, which suggests an important role of P2Y12 *in vivo*. A new class of oral antiplatelet drugs specifically targeting the P2Y12 receptor has been developed [24, 51, 53]. Ticagrelor was the first drug in this class that was shown to reversibly bind to the P2Y12 receptor. It effectively inhibits platelet aggregation and blood clot formation, thereby improving the outcomes of patients.

Thus, ticagrelor was noted as the first reversibly binding oral P2Y12 receptor antagonist. After discontinuation, the duration of its effects is shorter, and the risk of bleeding is lower than that of clopidogrel. Since ticagrelor does not require metabolic activation, its efficacy does not depend on P450 polymorphism. Therefore, it has better inhibitory effects on platelet aggregation. However, its half-life is very short, only 12 hours. Given that its effects disappear quickly after discontinuation, it has to be administered twice daily, necessitating strong patient compliance. Long-term use of ticagrelor may increase the risk of bleeding. Furthermore, patients with a history of asthma and heart failure may experience breathing difficulties during ticagrelor treatment; therefore, caution should be exercised when using this drug.

### 5.2 Rivaroxaban

Rivaroxaban directly inhibits fibrinogen (FXa) production, effectively preventing thrombosis. Unlike other anticoagulants, rivaroxaban does not rely on other factors for its antiplatelet activity. However, it is metabolized by P450 in the liver; therefore, its metabolism in the body is rapid, and frequent monitoring of the clotting index is not required [54]. In addition, rivaroxaban neither inhibits established thrombin activity nor interferes with normal clotting function.

Clinical studies have shown that low-dose rivaroxaban combined with aspirin has better therapeutic efficacy in patients with stable CHD. The treatment model can not only save medical costs but also improve the quality of life of patients [55]. Although rivaroxaban is a highly effective anticoagulant, it may increase the risk of bleeding in some patients. Therefore, to ensure safety, prevent bleeding, and achieve optimal treatment effects, rivaroxaban should be administered with extreme caution, and its advantages and disadvantages should be comprehensively analyzed for each patient.

### 5.3 Indobufen

The therapeutic effects of indobufen are different than those of traditional antiplatelet drugs. Aspirin exerts antiplatelet effects by blocking COX-1, thus preventing the conversion of AA to TXA2 and prostaglandin I2 (PGI2) [56, 57]. However, it leads to irreversible inactivation of COX-1. As the activity of COX-1 gradually recovers after discontinuing aspirin administration, it may lead to excessive platelet activation or other related complications [58, 59].

In contrast to aspirin, indobufen leads to reversible inactivation of COX-1 [60]. After discontinuing indobufen, its effects can be controlled, and no sustained or irreversible damage is caused. This property makes indobufen safer and more acceptable for clinical use, especially for patients who cannot tolerate aspirin or are at a high risk of bleeding [61].

In addition, indobufen does not significantly affect plasma coagulation parameters [62], suggesting that the clotting index does not change drastically even after long-term use of the drug. This characteristic is important because coagulation indicators are monitored regularly to evaluate the effectiveness of antiplatelet treatment. In addition, indobufen can rapidly restore platelet function to normal, thereby providing immediate safety benefits to patients.

### 5.4 Cilostazol

**Fig. 2.**
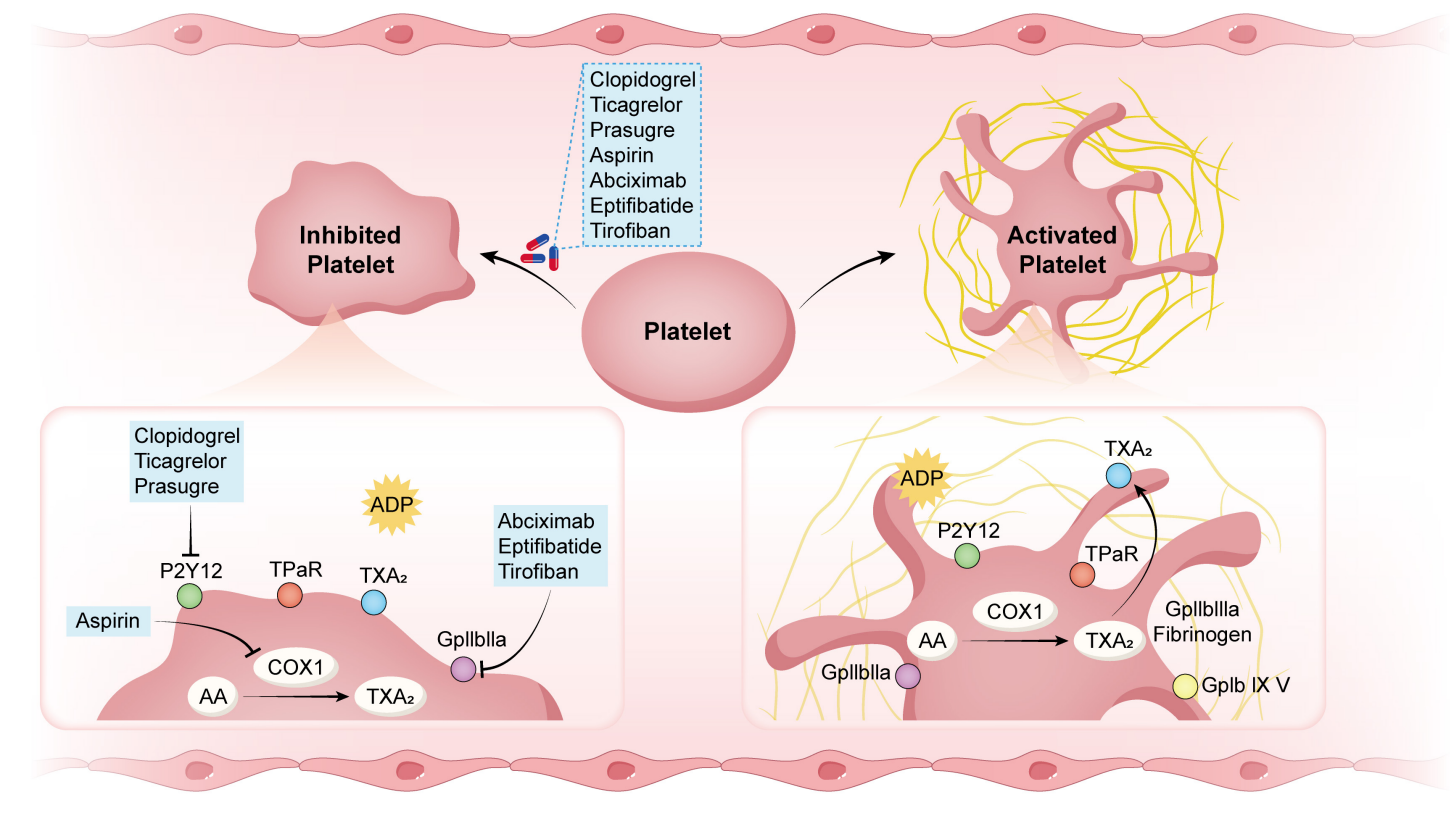
**Mechanisms of action of antiplatelet drugs**. ADP, adenosine diphosphate; AA, arachidonic acid; TXA_2_, thromboxane A2; GPIIbIIIa, glycoprotein IIbIIIa; GPIb IX V, glycoprotein (GP) Ib-IX-V; vWF, von Willebrand factor; COX-1, cyclooxygenase-1; TPαR, thromboxane A2α receptor.

Cilostazol, a phosphodiesterase inhibitor, can effectively inhibit platelet aggregation, vasoconstriction, and cell proliferation. Cilostazol is used to treat various diseases, such as peripheral artery disease, cerebrovascular disease, and CHD [63, 64, 65, 66, 67]. The 3,4-dehydro-cilostazol metabolite forms the main therapeutic component of cilostazol [68]. Cilostazol inhibits the activity of phosphodiesterase (PDE), increases intracellular cAMP content, prevents the synthesis of TXA2, and suppresses the secretion of ADP and 5-hydroxytryptamine by platelets, thereby exerting antiplatelet and vasodilatory effects [69]. It is widely used to treat intermittent claudication in clinical practice [70]. In addition to exerting potent antithrombotic effects, cilostazol possesses anti-atherosclerosis and anti-arrhythmia activities and protects vascular endothelial function (Fig. 2) [71, 72].

The above-mentioned antiplatelet drugs have different mechanisms of action, target populations, and application prospects. Notably, they are considered more effective treatment agents for CHD [73, 74]. Their specific classification and characteristics are summarized in Table 2 (Ref. [54, 57, 75, 76]).

**Table 2.** **Classification and characteristics of novel antiplatelet drugs**.

	Drug classification	Drug name	Characteristics
Receptor antagonists	Adenosine diphosphate receptor antagonist (ADPR-A)	Ticagrelor	It specifically targets the P2Y12 receptor [75].
Enzyme inhibitors	Phosphodiesterase inhibitor	Rivaroxaban	It is more effective and safer than aspirin and can significantly reduce the incidence of bleeding. It serves as an alternative to aspirin in patients who have a history of gastric ulcers or have difficulty tolerating aspirin-induced gastrointestinal reactions [76].
	Epoxide inhibitor	Indobufen	It reversibly inactivates COX-1 and can be used as an alternative to aspirin [54].
Others	Inhibition of FXa	Cilostazol	It inhibits FXa-induced platelet activation through the PAR-1 pathway [57].

FXa, fibrinogen; COX-1, cyclooxygenase-1; PAR-1, protease-activated receptor-1.

## 6. Selection of Antiplatelet Drugs

Owing to its irreversible inhibitory effects on platelet activation, aspirin is widely used for primary and secondary prevention of cardiovascular disease. The combined use of aspirin and clopidogrel bisulfate or ticagrelor has been validated as an effective treatment option for CHD in multiple large-scale clinical trials [77]. However, aspirin can affect prostaglandin production, directly damage the gastrointestinal mucosa, and increase the risk of gastrointestinal bleeding [78]. Moreover, some patients with CHD have poor tolerance to aspirin, which leads to more adverse reactions, such as nausea and vomiting, and limits the use of aspirin [79]. In particular, approximately 20% of patients are intolerant to aspirin, whereas 0.5%–2.4% of patients have allergic reactions to aspirin [80]. If P2Y12 receptor antagonists are used alone to inhibit platelet activation, the risk of thrombosis increases, increasing the risk of acute myocardial infarction and in-stent restenosis [81]. Indobufen, a reversible and highly selective inhibitor of platelet COX-1, blocks phospholipase A2 (PLA2), effectively preventing platelet aggregation without affecting the synthesis of prostaglandins (PGs) in the gastrointestinal mucosa [82]. Therefore, the risk of gastrointestinal bleeding is lower in patients receiving indobufen than in those receiving aspirin. In addition, indobufen has a faster onset of action and better absorption, and platelet function is rapidly recovered after its discontinuation [83]. Therefore, indobufen is a better option for patients with CHD with aspirin intolerance and a high risk of gastrointestinal bleeding [84]. However, for patients with CHD and atrial fibrillation complications, oral anticoagulant monotherapy (such as rivaroxaban) at stroke-prevention doses represents the first-line treatment option [85, 86]. For patients with a high risk of ischemia but a low risk of bleeding, aspirin at a dose of 75–100 mg/d or clopidogrel at a dose of 75 mg/d can be used as long-term oral anticoagulants [86]. Ticagrelor does not require metabolic activation, has a rapid onset of action, and exerts strong inhibitory effects on platelet aggregation; however, it increases the risk of major bleeding events [87]. Given the higher risk of bleeding in older patients, a comprehensive assessment of bleeding risk should be conducted when using ticagrelor, and its dosage should be adjusted accordingly. Ticagrelor and prasugrel are contraindicated for patients with active bleeding or a history of intracranial hemorrhage. Therefore, both drugs should be cautiously used in patients with a high risk of bleeding [88, 89].

Studies have shown that low body weight, heart failure, and peripheral arterial disease are high-risk factors for bleeding in East Asian populations [90, 91, 92, 93]. Furthermore, chronic kidney disease, low body weight (<60 kg), and advanced age (>80 years) are independent risk factors for bleeding [94]. Patients with diabetes have a high risk of ischemia and bleeding; however, they also have other risk factors. Therefore, whether diabetes represents a potential independent risk factor for bleeding warrants further verification [95]; interestingly, systemic lupus erythematosus can also increase the risk of bleeding owing to active thrombocytopenia and platelet dysfunction [96]. Thus, caution should be exercised, and the beneficial and harmful effects of antiplatelet drugs should be compared when administering these drugs to these patient populations.

## 7. Conclusions and Outlook

### 7.1 Conclusions

Preventing thrombosis is key to improving the prognosis of patients undergoing PCI. Clinical studies have shown that personalized antiplatelet treatment can reduce side effects, improve efficacy, and minimize treatment risks. Aspirin and ticagrelor can cause damage to the gastrointestinal mucosa, leading to a high risk of gastrointestinal bleeding. The gastrointestinal risk is low due to the reversible and highly selective inhibition of platelet aggregation by indobufen. Additionally, indobufen can improve red blood cell deformability and counteract coagulation factors, causing minimal damage to the gastric mucosa. Therefore, it has broad clinical application prospects, especially for patients with aspirin intolerance. However, existing studies on indobufen mostly have small sample sizes, and the results require further validation in larger cohorts.

For patients with CHD and a high bleeding risk, the duration of dual antiplatelet therapy (DAPT) should be shortened, and the type and dosage of antiplatelet drugs should be adjusted according to the bleeding risk. For older patients, the risk of bleeding should be closely monitored during treatment. Targeted preventive or treatment measures should be adopted based on their symptoms, comorbidities, cognitive function, and life expectancy. DAPT is not recommended for patients with short life expectancy, progressive malignant tumors, poor compliance, poor mental state, end-stage renal disease, advanced age, history of major bleeding/hemorrhagic stroke, long-term alcohol abuse, anemia, and history of severe bleeding during DAPT. For frail or older individuals, monotherapy with antiplatelet drugs should be prioritized. Individualized treatment dosages should be selected, and the benefit-to-risk ratio should be regularly or dynamically evaluated to adjust treatment strategies in a timely manner. In addition, medications should also be adjusted in a timely manner for patients with antiplatelet resistance.

### 7.2 Outlook

During antiplatelet therapy, attention should be paid to the risk of bleeding, and drugs should be rationally used according to the specific conditions of each patient. Patients should be screened for HTPR, and a personalized treatment plan should be designed to improve therapeutic efficacy while reducing the risk of side effects.
